# Understanding the Mechanism of Antimicrobial Resistance and Pathogenesis of *Salmonella enterica* Serovar Typhi

**DOI:** 10.3390/microorganisms10102006

**Published:** 2022-10-11

**Authors:** Maryam Khan, Saba Shamim

**Affiliations:** Institute of Molecular Biology and Biotechnology, The University of Lahore, Defence Road Campus, Lahore 54000, Pakistan

**Keywords:** *Salmonella*, typhoid fever, antimicrobial resistance, plasmids, pathogenicity islands, multidrug-resistant, extensively drug-resistant

## Abstract

*Salmonella enterica* serovar Typhi (*S. Typhi*) is a Gram-negative pathogen that causes typhoid fever in humans. Though many serotypes of *Salmonella* spp. are capable of causing disease in both humans and animals alike, *S. Typhi* and *S. Paratyphi* are common in human hosts only. The global burden of typhoid fever is attributable to more than 27 million cases each year and approximately 200,000 deaths worldwide, with many regions such as Africa, South and Southeast Asia being the most affected in the world. The pathogen is able to cause disease in hosts by evading defense systems, adhesion to epithelial cells, and survival in host cells in the presence of several virulence factors, mediated by virulence plasmids and genes clustered in distinct regions known as *Salmonella* pathogenicity islands (SPIs). These factors, coupled with plasmid-mediated antimicrobial resistance genes, enable the bacterium to become resistant to various broad-spectrum antibiotics used in the treatment of typhoid fever and other infections caused by *Salmonella* spp. The emergence of multidrug-resistant (MDR) and extensively drug-resistant (XDR) strains in many countries of the world has raised great concern over the rise of antibiotic resistance in pathogens such as *S. Typhi*. In order to identify the key virulence factors involved in *S. Typhi* pathogenesis and infection, this review delves into various mechanisms of virulence, pathogenicity, and antimicrobial resistance to reinforce efficacious disease management.

## 1. Introduction

*Salmonella enterica* serovar Typhi (*S. Typhi*) is a Gram-negative, rod-shaped, flagellated bacterium. The entire bacterium is covered with a capsule that is attributable to its virulence and evasion of phagocytosis in the host, thereby aiding in causing infection [1]. Various species of *Salmonella* are pervasively found in diverse environments and are capable of causing infection in humans and animals alike. These intracellular pathogens can evade and resist immune responses in the host, which can effectively trigger virulence and cause infection. Classified according to the White–Kauffmann–Le Minor scheme, approximately 1600 serotypes of *Salmonella* have been sorted into the subspecies *enterica* [2]. On a general scale, most serotypes are basically categorized into typhoidal and nontyphoidal strains, in which the former includes *S. Typhi* and *S. Paratyphi* A, which are common in humans only, while the latter strains are reported to infect both humans and several species of animals, resulting in different illnesses, such as enteric fever, sepsis, gastroenteritis, and salmonellosis, respectively [3].

Humans are the reservoir of *S. Typhi*, which has limited ability to multiply outside its host, though it may be capable of surviving for a prolonged time in the environment [4]. The mode of its transmission is largely indirect and most commonly vehicle-borne via contaminated food and water sources [5,6]. Though *S. Typhi* is capable of surviving for long periods of time in the environment, it does not multiply in food and water sources. The transmission of *S. Typhi* is classified into two patterns. Short-cycle transmission refers to the contamination of food and water sources by shedding of the bacterium through feces in the immediate environment or close proximity, which facilitates transmission through poor hygiene and sanitation practices, and is generally linked to food handlers [7]. Long-cycle transmission occurs when the broader environment (such as untreated water or sewage) is polluted with human feces or when it is used as a raw fertilizer for crops [8]. Long-cycle transmission pathways are difficult to trace because it is challenging to isolate *S. Typhi* from the environment [9]. The entry route of *S. Typhi* is the mouth, where the pathogen enters through ingesting food and water sources contaminated with fecal matter. Postinfection, the incubation period decreases while the risk of disease increases, correlating with the ingested dose [4,10,11]. *S. Typhi* is regarded as the primary cause of typhoid fever, usually contracted via the ingestion of contaminated food and/or water [12]. It is often considered a travel-associated disease [13,14]. In countries such as Pakistan, the causative pathogen is the major reported cause of pediatric septicemia [15,16,17]. Moreover, *Salmonella* spp. are globally reported to be major foodborne pathogens, accounting for <50,000 cases in Europe alone, with more than 80 million annual cases recorded for the year 2020 [18]. Meat, eggs, live poultry, and dairy products are recognized as great risk sources [19,20]. Moreover, the risk of infection is associated with various factors, predominantly poor hygiene and lack of clean water, as well as lifestyle and environmental hazards in middle- and low-income countries, while in high-income countries, the risk factors are mainly linked to quintessential contamination of fruit, vegetable, and meat sources [21,22,23].

Though there have been various advances in the practices of healthcare and medicine, an increasing number of people have been at risk of contracting typhoid fever, which can be a leading reason for death in serious or complicated cases. Prior to treatment with antimicrobial drugs, the fatality risk of typhoid fever fell between 10% and 30%, which has now reduced to less than 1%. Presently, the rise of resistant *S. Typhi* strains that evade antimicrobial agents poses a significant threat to its effective treatment [24]. The terminology “MDR” refers to “multidrug-resistant” strains, which are seemingly resistant to several broad-spectrum and first-generation antibiotics, whereas the term “XDR” refers to “extensively drug-resistant” strains, which are resistant to several antibiotics, such as ampicillin, fluoroquinolones, and chloramphenicol, along with many third-generation antibiotics, respectively [25]. According to antimicrobial resistance data (2018/2019) by EFSA, the prevalence of MDR *Salmonella* spp. in humans was reported to be more than 25% [26]. Though the fatality rate is less in developed countries, typhoid fever is a disease that still causes more than 200,000 fatalities annually worldwide [27]. There are several predisposing factors that indicate the severity of typhoid fever, which are necessary for the evaluation of its treatment, prevention, and mitigation. This information is critical in managing the economy of healthcare and its resources, which are important for the regulation of public health. Moreover, the required information and knowledge of the current trend of disease is very important with respect to frequent transmission [28].

## 2. Typhoid Fever: Epidemiology, Clinical Manifestations, and Diagnosis

Typhoid fever presents itself to be a cause of global concern, with more than 27 million cases and approximately 200,000 deaths every year globally [29,30]. While cases are recorded all over the world, the regions contributing most to the global burden are Africa, South and Southeast Asia, as well as the Western Pacific regions [31,32,33]. In Pakistan, the provinces of Punjab and Sindh are reported to be declared the most severely affected, among other Asian regions [34,35].

Typhoid fever is marked by potentially life-threatening fever with a multitude of clinical signs and symptoms. In spite of many years of research, much about the disease is still unknown [25]. Its first incidences are reported to date back to the early 19th century, bringing it close to the time when its causative agent, *S. Typhi*, known as typhoid bacillus at that time, was discovered by Dr. Karl Eberth in the 1880s [36]. It is usually characterized as a disease that typically afflicts children, immunocompromised adults, and the elderly, while the incidence of infection in infants happens to be rare but is present [37]. Most hospitals present cases affecting children and adults within the age bracket of 5–25 years [38,39]. The entry of *S. Typhi* and its subsequent invasion and attack on the host cells is accompanied by a short period of bacteremia, which presents no clinical symptoms in the host. This incubation period usually spans 2 weeks but can also last a month, a factor that is largely dependent on the bacterial count in host cells. Once this incubation period has passed, symptoms can comprise of fever, fatigue, loss of appetite, headache, myalgia, nausea, cough (dry), and diarrhea. Occasional shedding of bacteria through stool can be possible before the manifestation of any clinical symptoms of disease, which might include fever, abdominal discomfort and pain, rose-colored spotting on skin, and organ perforation [40]. In the case of insufficient or lack of treatment thereof, body temperature remains elevated, while symptoms such as nausea, increased pulse rate, headache, and persistent cough remain apparent [12]. These symptoms can highly vary from mild to severe, where mild symptoms are low fever, fatigue, and diarrhea, including perforations in the intestines in the case of acute and chronic inflammation, internal bleeding, and hemorrhage during infection [12,41]. Moreover, the intestinal surface surrounding the lesions can be comparatively healthy and unaffected compared with the affected area. Surprisingly enough, *S. Typhi* that can be cultured is not usually found at the perforation sites, though the DNA of the bacterium is often detected [42]. As part of the immune response, CD68+ macrophages are the most dominant immune cells at affected sites, along with B and T cells. Therefore, these perforations may be clinically and pathologically similar to the presensitization phase, as observed from “Shwartzman and Koch” reactions. Moreover, neurological complications may arise in the most severe of cases [43,44]. The clinical and pathophysiological manifestation of typhoid fever is largely dependent upon the severity of the case in patients. In regions with a high incidence of the disease, community-based investigations have suggested that many of the patients suffer from atypical typhoid fever [45]. Therefore, an approximate number of 60–90% are treated as outpatients in various hospitals. In the case of patients receiving hospital care, sufficient care, good nutrition, careful administration of antibiotics regimen, as well as the prevention of disease complications are the major practices that need to be followed to avoid complications and fatality associated with the disease [16,25].

For the diagnosis of typhoid fever, the standard procedure remains to be bone marrow culture, but it is not commonly pursued, as it is not practical to perform it in several endemic-hit areas. Instead, blood culture is more commonly adopted as a customary procedure for diagnosing typhoid and paratyphoid fever, which stands at an average sensitivity of more than 6%, according to a review [46]. Therefore, the method of diagnosis stands to be a crucial point in effective treatment and is very important to develop new, efficient, safer, and cheaper methods for swift diagnosis, which can be essential in saving lives in critical cases [47]. Moreover, rapid diagnostic tests could be used in combination with clinically devised algorithms for the differentiation of febrile patients and chronic carriers for a more directed approach toward effective management, particularly in areas where there is a dearth of sufficient laboratory equipment and medical facilities. Many rapid diagnostic tests such as Typhidot, Typhidot-M test, TUBEX, and Test-It have been developed for the swift diagnosis of typhoid, paratyphoid, and enteric fever. However, these tests are less sensitive for disease detection [48]. Other diagnostic methods that are being developed for the detection of pathogens include antibody in lymphocyte (ALS) supernatant, which has been reported to exhibit remarkable sensitivity and specificity levels in various endemic-hit areas [49,50,51]. In addition, PCR-based methods also show promise at a small scale, but no such method is in current use at a widespread level, due to which its effective sensitivity and specificity cannot be deciphered accurately. Nevertheless, this can be enhanced by the incorporation of a pre-enrichment step in the PCR-based assays [52]. However, widespread usage of molecular-biology-based methods is, therefore, limited by restricted medical and laboratory resources, the cost of the procedures, and the time period required to complete the procedure [53]. Future aspects of serovar detection can be based on high-throughput methods that can be swiftly utilized for the detection of pathogenic and resistant serovars through techniques such as mass spectrometry, antigen arrays, and next-generation sequencing (NGS) [54,55]. The use of mass spectrometry has been reported to be used in samples for the identification of typhoid fever from paratyphoid and enteric fever with the help of metabolites [56,57,58]. Moreover, information pertaining to transcription from patients suffering from typhoid fever (acute) may be employed for the identification of specific signatures, which can effectively aid in the detection of typhoid cases [59,60].

## 3. Emergence of Antimicrobial Resistance and Current Predomination Worldwide

Prior to the routine use of antibiotics for the treatment of bacterial infections, typhoid fever presented itself to be a great challenge in terms of effective detection and treatment. In 1947, the main antibiotic that was prescribed for treating typhoid and enteric fever was chloramphenicol, but the later years of the decade saw the emergence of resistance in *Salmonella* spp. As an outcome of increasing plasmid-mediated resistance against chloramphenicol, the majority of infections all over the world were now being caused by chloramphenicol-resistant *Salmonella* spp., which culminated in sporadic epidemics worldwide. An example would be the epidemic in Mexico, where more than 10,000 cases of typhoid fever caused by MDR strains were reported. Though that epidemic was controlled, other reports of breakouts in various countries were vastly reported in the coming years [35]. The past few decades have seen the rise of multidrug-resistant (MDR) *S. Typhi*, resistant to typical first-line antibiotics such as chloramphenicol, ampicillin, and trimethoprim/sulfamethoxazole, which has led to the use of fluoroquinolone antibiotics, such as ciprofloxacin, as first-line treatment. However, fluoroquinolone resistance, particularly in South Asia, has resulted in third-generation cephalosporins (e.g., ceftriaxone) being used as a first-line treatment [61].

Since the emergence of these MDR strains in the 1980s [62], the world has been observing the silent yet swift rise of these strains in terms of resistance, first with second-generation antibiotics such as fluoroquinolones. Since then, third-generation antibiotics such as cefoperazone, cefotaxime, and ceftriaxone, along with cephalosporins, have been employed for the treatment of *Salmonella* infections [63]. However, resistance against these antibiotics has been sporadically reported [64]. Despite the prevalence of MDR strains in Asian countries, cases have also been reported in regions of Africa, including South Africa, Egypt, Nigeria, and Kenya [65], as well as several countries across Europe [66]. In 2016, the city of Hyderabad, Pakistan, reported the first case of XDR *S. Typhi* [67], the number of which has escalated to more than 10,000 XDR cases in Pakistan alone, as documented by WHO [68]. Alarmingly, several cases of XDR *S. Typhi* have also been reported in developed countries such as Canada [69], Australia [70], Denmark [71], and the USA [72]. Likewise, outbreaks have also been reported in various other countries such as Bangladesh, the Philippines, Iraq, and India [73]. Moreover, cases of emerging antibiotic resistance have been reported to be exacerbated by international travel, enabling the transfer of resistant bacterial strains [74]. Furthermore, reports from early to late 2018 (January–October) indicated the spread of *S. Typhi* XDR strains to be associated with international travel to and from Pakistan, in which out of six reported cases, five were reported to originate from the United States (USA), while the remaining one was reported from the United Kingdom (UK). Initial reports revealed that four out of six cases were either permanent residents or had arrived (prior to infection) in Karachi, Islamabad, and Lahore. In the other cases, one patient was revealed to be a resident of Lahore, while the other had a travel history to Sindh (Karachi). Evidential data suggested that all travel-related cases of XDR strains were positively treated with effective treatment, but the route of exposure and the early onset was unknown. Recently, a case in Canada was treated for XDR typhoid, which originated in Pakistan, as the patient (a child) had traveled from Canada to Sindh, Pakistan, and then to Canada back again, where subsequent diagnosis and treatment were given [69]. During times of the COVID-19 pandemic, typhoid cases have been on the increase in Pakistan, with more than 20,000 cases being reported in June 2020 [75]. Over the past two decades, the emergence of *S. Typhi* haplotype H58, also known as 4.3.1, has been reported to be dominant in Asia and Africa [76]. This genotype has been associated with decreased *S. Typhi* susceptibility to fluoroquinolones [77], as well as the acquisition of IncHI1 plasmids for resistance. Upon phylogenetic analysis, it was found that this genotype might have originated from South Asia, but this is not well understood [78].

Conventionally, typhoid fever caused by MDR strains exhibits resistance to all first-generation antibiotics recommended by WHO, such as ampicillin, chloramphenicol, and sulfamethoxazole/trimethoprim. The rise of MDR and quinolone-resistant *S. Typhi* strains signifies a grave health risk in Pakistan, as quinolone-resistant strains are commonly reported in cases of typhoid and enteric fever. However, a previous study carried out (2001–2006) at Aga Khan University, Karachi, Pakistan, revealed that the incidence of MDR *S. Typhi* strains had increased from <30% to <45% [79]. The rise of quinolone-resistant *S. Typhi* strains has shifted treatment options to third-generation antibiotics (cephalosporins). In Sindh, resistance to third-generation antibiotics, including ceftriaxone, has been on the rise since 2016. This is proven true by the ability of *Salmonella* serovars to transform from MDR to XDR by acquiring a resistance plasmid, enabling resistance of the pathogen to antibiotics [80]. Moreover, the increasing resistance in *S. Typhi* against azithromycin [81,82,83] and meropenem has been reported in Pakistan and Indonesia [84,85].

## 4. Mechanisms of Antimicrobial Resistance in *S. Typhi*

In *Salmonella* spp., particularly *S. Typhi*, antimicrobial resistance could either be mediated by plasmid or chromosomal DNA [86]. Usually, resistance is developed by the inactivation of antibacterial agents and alteration of drug targets, as well as by employing various efflux pumps [87]. Genes expressing drug degradation enzymes and/or efflux pumps may be expressed via point mutation, or external factors of resistance may be actively mediated by gene transfer using virulence plasmids, phages, and mobile genetic elements [88].

### 4.1. Virulence Plasmids and Plasmid-Mediated Antimicrobial Resistance in S. Typhi

*S. Typhi* typically has plasmids that contain several virulence and antimicrobial resistance genes. These plasmids vary from 50 kb to 90 kb in size and carry the *spv* operon, which is significantly involved in causing infection [89], as the genes of this operon are reportedly pivotal for bacterial proliferation in host cells and supposedly enhance the virulence of the pathogen [90]. Though most virulence plasmids are not self-transferable, some of them do contain *tra* genes that enable the transfer of plasmids via conjugation [91]. Incompatible (Inc) plasmids are responsible for encoding multiple antimicrobial resistance in *S. Typhi* and are classified into IncH1, IncH2, and IncH3. Plasmids R27, pHCM1, and pAKU1 comprise a composited transposon that can harbor multidrug resistance in MDR *S. Typhi* strains [92]. The H58 clade is primarily attributable to MDR and XDR outbreaks, in which XDR strains harboring IncHI1 plasmid enable fluoroquinolone resistance to strains [93].

### 4.2. Antimicrobial-Resistance-Associated Genes in S. Typhi

The resistance to antibiotics in Gram-negative bacteria is primarily attributed to the production of *β*-lactamases (ESBLs). TEM, SHV, and CTX-M are the main types of ESBLs in *Salmonella* spp., which confer resistance to penicillin and cephalosporin [94]. These enzymes are primarily antimicrobial degrading enzymes, which do so by cleaving the β-lactam ring. In *S. Typhi*, the presence of these genes has been attributed to the genetic transfer of resistance genes from other Gram-negative bacterial species and also selection pressure driven by the misinformed use of broad-spectrum antibiotics [95]. Tetracycline-resistant genes (*tetA, tetB, tetG*) are responsible for encoding resistance against tetracycline by activating the efflux pump responsible for transporting the drug out of the cell, thereby reducing its concentration. Genes conferring resistance against quinolones (*qnrA, qnrB, qnrC, qnrS*) encode for pentapeptide proteins, which offer bonding and protection to DNA gyrase and other enzymes. The *cat1* and *cat2* genes mediate resistance to chloramphenicol by inactivating it through the action of the acetyltransferase enzyme. Genetic elements identifying the mobile gene cassettes that carry multidrug-resistant genes are known as integrons. In *S. Typhi*, the presence of integrons (class 1 and 2) equalizes the distribution of antimicrobial resistance, in which class 1 is more dominantly found [96,97,98].

## 5. Pathogenesis of *S. Typhi*

The infectious dose of *S. Typhi* in humans has been observed to be more than 10,000 cells in order to cause active infection, but this figure is subject to change in terms of various host and environmental factors. Enteropathogenic pathogens weaken the gut epithelia by exploiting tight junction (TJ) components for invading gut cells or tissues or promoting signaling responses that augment their invasion [99,100]. *S. Typhi* invasion of the gut epithelia is reported to increase TJ permeability [101]. Furthermore, there have been studies that suggest a lower count of the bacterium [102,103]. The pathogen tends to attack the mucosal lining of the intestines via the action of microfold cells (M cells), thereby helping to form an undetectable bacterial load in the absence of clinical signs and symptoms, resulting in general bacteremia. Therefore, the bacterium is able to invade the host system but does not necessarily trigger the onset of a rapid immune host response. This is a vital aspect of *S. Typhi* infection, in which the main inflammatory response is lacking in the host and is different from the infection caused by nontyphoid serovars of *Salmonella* spp. [104]. It has been found that the ability of gut mucosal penetration correlates with the ability of the bacterial species to invade nonphagocytic cells by expression of a type III secretion system (T3SS), known as T3SS-1. When *Salmonella* spp. reach the small intestine, the expression of SP-1 is induced by several stimuli, such as elevated osmolarity and iron concentration, neutral pH, and decreased O2 levels [105]. T3SS-1 enables the injection of bacterial effector proteins directly into the host cells, promoting actin polymerization and ruffling membrane rearrangements, thereby leading to bacterial internalization. This mechanism is known as the trigger mechanism and is widely dependent on host cells [106]. Postinfection, the incubation phase of the pathogen may not always be demonstrated by a symptomatic phase.

The understanding of the pathogenesis, replication, and transmission of a particular pathogen is very crucial for its diagnosis, treatment, and prevention. This aspect is now elucidated better due to the breakthrough advances in genomics and molecular biology, which aid greatly in deciphering the behavior of a pathogen as well as its mode of transmission. The pathogenesis and the response of the host against *Salmonella* infection are dependent upon the attachment and ability of the pathogen to invade the host’s epithelial cells, after which it can effectively disseminate to surrounding sites via the action of phagocytic cells. The final stage of the infection comprises the survival, replication, as well as transfer of the pathogen from one host to another, thereby initiating active transmission among susceptible hosts. *S. Typhi* enters the human host through contaminated sources of food and water and tends to pass through the stomach into the epithelial cells of the gut. The first challenge of *Salmonella* colonization is stomach acidity, and certain situations in which it either is reduced (by the usage of antacids, proton pump inhibitors) or the intestinal integrity is compromised (surgery, antibiotic use, inflammatory bowel disease) elevate the chances of *Salmonella* infection in the host. This is mediated by the adaptive acid response of *Salmonella* spp. upon acid exposure *in vitro*, which probably facilitates survival and movement into the small intestine. As in the case with most pathogens, attachment of bacterial cells is pertinent prior to their invasion in the host, which is dependent upon the adhesion molecules of the bacterium, which then interact with the host receptors. This interaction is not elucidated well but is assumed to be facilitated by fimbrial adhesion present in the outer region of the bacterial cell. It has been reported that *S. Typhi* possesses 12 fimbriae regulating operons of chaperone class, but none of these are particularly unique to *S. Typhi* [107]. This diversity may be attributable to the selective response by the host [108]. For instance, type IV-B pilus operon (*pil*) is expressed in *S. Typhi*, which enables wild-type (*pil+*) *S. Typhi* strains to mediate adherence to host intestinal cells [109]. The evasion of intestinal epithelial cells by *Salmonella* spp. is mediated by endocytosis, which involves rearrangement of the cytoskeleton, disrupting the epithelial cell border as well as the reconfiguration of membrane ruffles. Under conditions similar to human intestinal cells, many efficiently adhering species of *Salmonella* are activated for the invasion of host cells. These species are regulated partly by the pathogenicity island (SPI-1) of *Salmonella* and its encoded regulatory and effector proteins, which altogether cause alterations within the host cells, thereby easing pathogenesis. The induction of SPI-1 by ancestral strains of *S*. *enterica* undoubtedly enables the efficient adherence and invasion of epithelial cells, which allows for the colonization of a new host environment [110].

*S. Typhi* is able to cause systemic infection in human hosts. This infection is often restricted to eventually generating a secretory immune response in the human intestine and its epithelium, where immune cells, particularly neutrophils, are secreted [111,112,113]. Moreover, it also enables the secretion of interleukin-8 (IL-8) along with other chemoattractants commonly induced by the presence of pathogens in the intestinal epithelium, which then direct the migration of neutrophils into the affected region [114]. In the absence of migration to and the presence of neutrophils in the gut region, *S. Typhi* may be able to invade and attack more invasively than before, though previously, this theory was not supported by enough evidence. Nevertheless, it has long been reported that *S. Typhi* does, however, stimulate the secretion of interleukin-6 (IL-6) in epithelial cells [115]. Though it has been established worldwide that *S. Typhi* causes a more aggressive form of the disease when compared with *S. Paratyphi*, these data may differ, as a study conducted in Nepal suggested that both pathogenic serovars caused similar clinical symptoms, thus causing equal or comparably identical forms of the disease in the tested subjects [116]. Around 5% of acutely infected patients tend to become chronic carriers of the pathogen when there is a lack of efficacious antimicrobial treatment. Chronic carriage comes with its own risks and adds to the burden of disease by mediating its persistence, thereby complicating treatment and mitigation practices [117]. Therefore, efforts to control the spread of disease in the future must be circled around identifying and treating chronic carriage of pathogens [118].

## 6. Pathogenicity and Its Role in *S. Typhi* Virulence

### 6.1. Salmonella Pathogenicity Islands (SPIs)

The capability of *S. Typhi* infection is dependent on virulence genes, which are ultimately located in *Salmonella* pathogenicity islands (SPIs), which are distinct genetic components (acquired from other pathogenic bacteria via horizontal gene transfer) found on chromosomal regions of pathogenic bacteria [119]. These SPIs carry a base composition distinct from the core genome, which is why they are often associated with mobile genetic elements and tRNA. Recent research has elucidated about 15 SPIs in *S. Typhi*. Several virulence factors known for adhesion, invasion, and toxins are found to be clustered in the SPIs [120]. Virulence genes have also been reported to be associated with SPIs, in which they serve various functions such as pathogen survival, bacterial multiplication, and evasion of host immune responses, respectively. SPI-1 to SPI-10 and SPI-15 to SPI-17 are all reported SPIs for *S. Typhi* (Table 1). Among these, SPI-1 and SPI-2 are the most commonly studied and primarily contribute to pathogenicity, as they encode the type III secretion system of proteins. This system of proteins is directly involved in the host–pathogen interaction during pathogenesis, which makes it pivotal to be encoded on SPIs [121]. The host macrophages and their interaction with *Salmonella* lead to the altered expression of a number of genes comprising pro- and anti-inflammatory mediators as well as adhesion receptors [122]. Some other genes that are regulated are those that encode proteins involved in cellular death [123,124]. Some particular serovars different from *S. Typhi* are reported to facilitate the induction of sudden and acute macrophagic death in a very short span of time (approximately 30 min) postinfection [125], which is regulated by the effector protein SipB of SPI-1, contingent on the cellular protein of the host cell (caspase-1) [126,127]. This protein is significant in mediating apoptosis through proinflammatory mediators, which is a counter-attack measure on the part of the host cells in case of a systemic infection in the host [128]. This proinflammatory action and the eventual recruiting of the phagocytic immune cells may contribute to bacterial dissemination. Host cell cytotoxicity promoted by *Salmonella-*induced caspase-1 might take place independently or in association with the activation of caspase-2, which acts as an initiator of caspase-1 [129].

Proteins associated with initial adhesion and survival during systemic infection are encoded by SPI-3, a conserved SPI, allowing *S. Typhi* and other *Salmonella* spp. to persist in environments where nutrition is scarce [130]. SPI-4 ensures intracellular survival in host macrophages and also supposedly carries a type I toxin secretion system [131]. SPI-5 is associated with encoding effector proteins (*sopB* gene) for both types of type III secretion systems [132]. SPI-7 encodes for Vi antigen, while genes for putative virulence factors and resistance to bacteriocins are encoded by SPI-8 [130]. SPI-9 is responsible for encoding the type I secretion system and its proteins, whereas SPI-10 encodes for fimbriae virulence factors, such as sef fimbriae. SPI-11 to SPI-14 are not reportedly found in *S. Typhi*, while SPI-15 serves a vague role with effector proteins attached to secretion systems. Genes and proteins for tRNA and lipopolysaccharide are encoded by SPI-16 and SPI-17, respectively [133].

### 6.2. Vi Antigen

Vi capsular polysaccharide (Vi) is a significant virulence factor of *S. Typhi* and is encoded by the B locus and is essential for the biosynthesis of the capsular part of the antigen. In the case of this capsule being present, *S. Typhi* is comparatively more invasive and lethal in attacking host cells in serum than other serovars of *Salmonella* [134]. The capsular part of the Vi antigen exhibits potential immunomodulatory properties that contribute to pathogenesis, disease progression, inhibition of bactericidal activity, reduction in the host immune response, and limitation of the complement deposition. This important aspect is why the Vi capsule has been considered to be contained as an integral part of traditional and conjugate typhoid vaccines. Apart from *Salmonella* spp., other significant bacterial pathogens also express the Vi antigen, such as *Citrobacter* spp. [135]. Moreover, the genome of *S. Typhi* contains various pseudogenes that have been observed to depict the inhibition of the host and its immune responses by the pathogen in an identical process previously reported in other similar pathogens [136]. *S. Typhi* is also reported to contain more than 300 genes that are only specific to this serovar. This genetic specificity is identified by the findings of an additional exotoxin known as the typhoid toxin, which has a significant role in driving the pathogenesis of enteric fever caused by *S. Typhi* and Paratyphi A. Therefore, it is important to characterize virulence factors that may serve a vital role in the pathogenesis of infection, which could then be investigated for the development of vaccines [53].

## 7. Conclusions

While regional outbreaks can be unprecedented, travelers arriving in Pakistan and other affected countries appear to be at high risk of MDR and XDR typhoid infection; consequently, a sufficient amount of health strategies and prevention steps are recommended and advised by the CDC to be taken for efficient and safe travels. Vaccines for use against typhoid have been reported since the development and widespread usage of heat-inactivated whole-cell vaccines in the latter half of the 19th century. However, it was not until now that the efficacy has been reported to be limited. Vi-polysaccharide vaccine, along with a live attenuated vaccine, provides 60–80% of protection, but one of its major downsides is that it requires readministration every 24–36 and 60 months, respectively. Moreover, none of the vaccines are recommended for use in children less than 24 months, which makes typhoid vaccination arduous to be incorporated into their vaccination programs, especially in underprivileged countries. Furthermore, new drugs could be designed to curb the resistance to antibiotics, along with proper monitoring of drug use, prescription, and awareness of usage, which could eventually decrease the global burden of *S. Typhi* infection and the occurrence of MDR and XDR strains.

## Figures and Tables

**Table 1:** Description of major *Salmonella* pathogenicity islands (SPIs) and their associated effector proteins.

SPIs	Protein	Function
SPI-1	SipA SipC	Promote membrane ruffling and *Salmonella* invasion by directly interacting with actin cytoskeleton
	SopE SopE_2_	Promote membrane ruffling and *Salmonella* invasion by directly interacting with actin cytoskeleton by inducing membrane ruffling after injection into epithelial cells
	SipB	Nucleates actin and translocates other effector proteins
	IaeP	Involved in post-translational modification of T3SS
	SopA SopC	Recruit immune cells and secrete fluid in intestinal lumen
	AvrA	Inhibits cellular apoptosis Inhibits macrophage degradation
	SicA SicP	Serve as a chaperone
SPI-2	SsaB	Disrupts Golgi apparatus
	SpiC	Disrupts vesicular transport
	SsaE SscA	Serve as a chaperone
	SsPH2 SseJ	Rearrange cytoskeletal system
	SrFT	Cellular apoptosis
	PipB SopD_2_	Target pathogen-induced filaments
SPI-3	MgtC MgtB	Ensure adaptation in nutrition-scarce environment
	MisL	Ensures attachment to epithelial cells
	MarT	Activates MIS L protein
SPI-4	SicE	Ensures attachment to epithelial cells
SPI-5	SsrAB	Serves key role in developing infection
SPI-6	Invasion proteins	Ensure invasion of pathogen into host cells
SPI-7		Produces Vi antigen
SPI-8		Contributes to putative virulence
SPI-9	T1SS and RTX	Contribute to toxin production and invasion of pathogen into host cells
SPI-10		Production of sef fimbriae
SPI-11		Function not clear
SPI-12		Function not clear
SPI-13		Function not clear
SPI-14		Function not clear
SPI-15		Serves a vague role to effector proteins attached to T3SS
SPI-16		Encodes genes for tRNA arg and lipopolysaccharides
SPI-17		Encodes genes for tRNA arg and lipopolysaccharides

## Data Availability

Not applicable.
